# IκB-Kinase-epsilon (IKKε) over-expression promotes the growth of prostate cancer through the C/EBP-β dependent activation of IL-6 gene expression

**DOI:** 10.18632/oncotarget.11629

**Published:** 2016-08-26

**Authors:** Benjamin Péant, Sophie Gilbert, Cécile Le Page, Alexis Poisson, Emilie L’Ecuyer, Zied Boudhraa, Marc Nicolas Bienz, Nathalie Delvoye, Fred Saad, Anne-Marie Mes-Masson

**Affiliations:** ^1^ Centre de Recherche du Centre Hospitalier de l’Université de Montréal (CRCHUM)/Institut du Cancer de Montréal, Montreal, Canada; ^2^ Department of Surgery, Hôpital Saint Luc (CHUM), Montreal, Canada; ^3^ Department of Surgery, Université de Montréal, Montreal, Canada; ^4^ Department of Medicine, Université de Montréal, Montreal, Canada

**Keywords:** IKKε, C/EBP-β, IL-6 gene promoter, prostate cancer, cell proliferation

## Abstract

The inflammatory cytokine IL-6 has been shown to induce the nuclear translocation of androgen receptors in prostate cancer cells and to activate the androgen receptors in a ligand-independent manner, suggesting it may contribute to the development of a castrate-resistant phenotype. Elevated IL-6 serum levels have also been associated with metastasis-related morbidity in prostate cancer patients. We have previously established that over-expression of I-kappa-B-kinase-epsilon (IKKε also named IKKi or IκBKε) in hormone-sensitive prostate cancer cell lines induces IL-6 secretion. We have also reported that prostate cancer cell lines lacking androgen receptor expression exhibit high constitutive IKKε expression and IL-6 secretion. In the present study, we validated the impact of IKKε depletion on the *in vitro* proliferation of castrate-resistant prostate cancer cells, and characterized how IKKε depletion affects tumor growth and IL-6 tumor secretion *in vivo* through a mouse xenograft-based approach. We observed a significant growth delay in IKKε-silenced PC-3 cells injected in SCID mice fed with a doxycycline-supplemented diet in comparison with mice fed with a normal diet. We also found a decrease in IL-6 secretion levels that strongly correlated with tumor growth inhibition. Finally, using constructs with various IL-6-mutated promoters, we demonstrated that IKKε over-expression induces a NF-κB-independent stimulation of the IL-6 gene promoter through the activation and nuclear accumulation of the transcription factor C/EBP-β. Our study demonstrates the pro-proliferative role of the oncogene IKKε in castrate-resistant prostate cancer cell lines, involving the phosphorylation and nuclear translocation of C/EBP-β that initiates IL-6 gene expression.

## INTRODUCTION

Epidemiological studies suggest a possible increased risk of developing prostate cancer (PC) in men with a prior history of chronic prostatitis (inflammation of the prostate gland) [1]. Among other events, inflammation is controlled by cytokine and chemokine release [2, 3]. In the tumor microenvironment, tumor-infiltrating immune cells and tumor cells themselves secrete pro-inflammatory cytokines [4]. Significantly elevated levels of the pro-inflammatory cytokine IL-6 have been observed in the serum of patients with castrate-resistant (CR) disease compared to patients with hormone-sensitive (HS) PC and high IL-6 expression (more than 7 pg/ml) is associated with PC progression and poor prognosis [5, 6]. There is also evidence suggesting that IL-6 has a growth-promoting role in PC by acting as a positive growth factor for most PC cells [7]. Moreover, IL-6 has been shown to induce transcription of the androgen-receptor (AR) gene, and to activate the AR in a ligand-independent manner [8–12]. These observations support the hypothesis that IL-6 acts as a key mediator of PC progression, particularly in the transition from HS PC cells to a CR state.

I-kappa-B-kinase-epsilon (IKKε), also named IKKi or IKBKE, is a lesser-known member of the IKK protein family and has been previously associated with the lipopolysaccharide-mediated secretion of IL-6 by murine embryonic fibroblast [13]. IKKε is traditionally linked with an inflammatory response and more specifically with the rheumatoid arthritis inflammation phenotype [14]. IKKε, together with the ubiquitous protein Tank Binding Kinase 1 (TBK1), forms a cytosolic heterodimer involved in Toll-Like Receptor 3 (TLR3)- and TLR4-mediated interferon production [15, 16]. Previously, we demonstrated that NF-κB is an important mediator of IKKε expression in PC [17]. Our research also revealed that CR PC cells exhibit high constitutive IKKε expression as compared to HS PC cells [17]. Moreover, IKKε over-expression in HS 22Rv1 and LNCaP cell lines induces the secretion of IL-6, and IKKε knockdown in CR PC-3 cells correlates with a significant decrease in their constitutive IL-6 secretion [18]. Subsequently, high levels of IKKε were observed in 30% of primary human breast cancers, suggesting oncogenic properties for this protein [19, 20]. IKKε deregulation has also been associated with the progression of ovarian cancers [21]. Recently, we studied the biological link between IKKε expression and PC progression *in vivo* using immunohistochemistry staining on a formalin-fixed paraffin-embedded primary PC tissue microarray [22]. We identified an increased IKKε expression during PC progression, when comparing non-malignant tissues to PC. Higher levels of IKKε expression in tumors correlated with the development of bone metastases and progression to CR PC in patients.

Although the constitutive expression of IKKε in CR PC is associated with inflammatory PC progression, little is known about the mechanism by which IKKε is involved in the progression of CR PC. In the present study, we characterized the direct molecular mechanism by which IKKε induces the secretion of IL-6 and investigated the impact of IKKε knock-down on the development of CR tumors *in vivo*.

## RESULTS

### Effect of IL-6 siRNA on PC-3 cell growth in serum free medium

**Figure 1:**
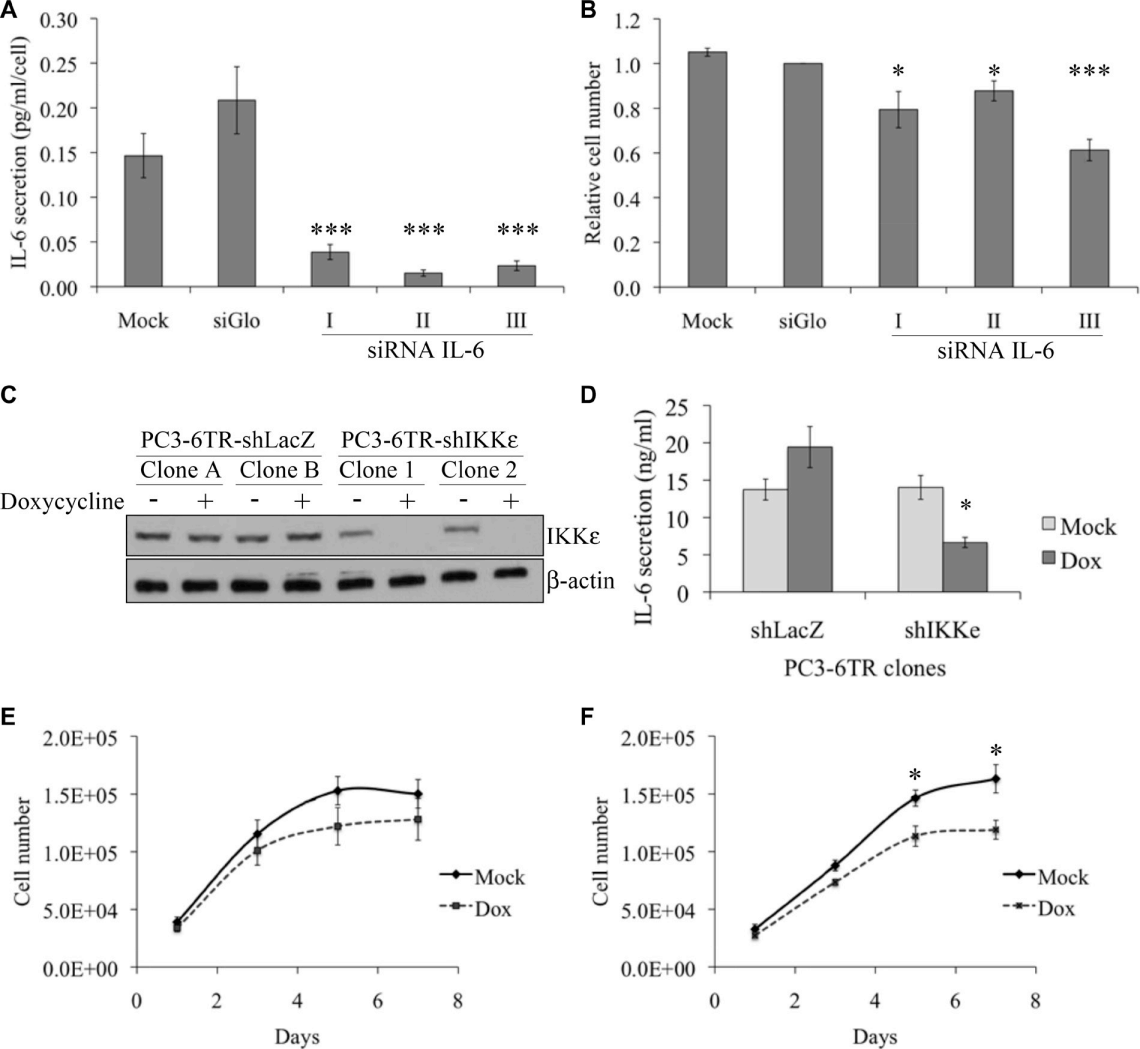
Effect of IKKε expression on IL-6 secretion and proliferation of prostate cancer cell lines (**A**–**B**) Impact of IL-6 knock-down on PC-3 proliferation. 60 000 PC-3 cells were cultured in low serum conditions in 12-well plates and transfected with control siRNA (si-Glo) or three different IL-6 siRNAs (5 μM). Five days after transfection, IL-6 secretion was quantified by ELISA (A) and live cells were quantified by trypan blue (B). (**C**) Characterization of IKKε expression in four independent PC3-6TR clones. Cells were grown in the presence or absence of doxycycline (50 ng/ml) for 40 hr. Whole cell extracts from PC3-6TR-shLacZ and PC3-6TR-shIKKε slones were separated by SDS-PAGE, transferred onto PVDF membranes and probed with anti-IKKε antibody. Equal loading was tested with an anti-beta-actin antibody. (**D**) Influence of IKKε expression on IL-6 secretion in stable, inducible PC3-6TR-shLacZ/shIKKε clones. IL-6 secretion was measured using ELISA performed on supernatants following 40 hr of culture with (black box) and without (gray box) doxycycline (Dox). Sample concentration was calculated using standard curves and was adjusted for 1 ml of cell culture supernatant and 1 mg of total cell proteins. (**E**–**F**) Effect of IKKε depletion on PC3-6TR-shLacZ (E) and PC3-6TR-shIKKε (F) cell growth in low-serum medium. Each measurement was done in triplicate and each experiment was repeated at least three times. Error bars represent the standard error of the mean. *P < 0.05; ***P < 0.0001 (paired *t-test*).

Since IL-6 is described as a positive growth factor for most prostate cells, and anti-IL-6 antibodies have an inhibitory growth effect on the PC-3 cells and xenograft derived from this cell line [7, 23], we wanted to confirm the autocrine role of IL-6 on PC-3 cell growth using an RNA interference strategy. IL-6 siRNA efficiency was validated by ELISA; IL-6 secretion in supernatants from PC-3 cell culture was quantified five days after transfection with three different IL-6 siRNAs or control siRNA (siGlo Green Transfection Indicator from Dharmacon) (Figure 1A). While the control siRNA appeared to induce an increase of secretion, each IL-6 siRNA induced a dramatic decrease of IL-6 secretion as expected. Five days after transfection, growth of PC-3 cells in serum-free medium was also significantly reduced by each IL-6 siRNA [from 12% with IL-6 siRNA-II (*P* = 0.034, *t-test*) to 37% with IL-6 siRNA-III (*P* < 0.001, *t-test*)] compared to siGlo (Figure 1B).

### Role of IKKε in IL-6 secretion and cell proliferation in prostate cancer cells

To analyze the role of IKKε on the regulation of IL-6 expression, we used PC3-6TR-shIKKε cells, which were engineered to have an inducible down-regulation of IKKε expression [18], and compared these cells with their respective control, PC3-6TR-shLacZ (Figure 1C). We noted that IKKε expression in unstimulated PC3-6TR-shIKKε clones was slightly lower than the expression observed in PC3-6TR-shLacZ clones. This observation suggests that our inducible system was partially leaky. We observed a 50% decrease (*P* = 0.01, paired *t-test*) in IL-6 secretion after a complete knock-down of IKKε expression in PC3-6TR-shIKKε cells that were cultured for three days in doxycycline-supplemented media (Figure 1D). Knock-down of IKKε expression also induced a decrease in PC3-6TR-shIKKε cell proliferation *in vitro*, particularly after five days of culture (*P* = 0.009, paired *t-test*; Figure 1E and 1F). This effect was completely abrogated when exogenous IL-6 was added in the culture media (Supplementary Figure S1).

We also observed a slight but non-significant increase of IL-6 secretion (*P* = 0.208, paired *t-test*, Figure 1D) in PC3-6TR-shLacZ cells cultured in doxycycline-supplemented media. This was associated with a non-significant decrease of cell proliferation (Figure 1E). These unexpected weak effects may be due to a general side effect that has been previously reported for the doxycycline used at our working concentration [24–26].

### Effect of IKK*ε* depletion on tumor growth *in vivo*

**Figure 2:**
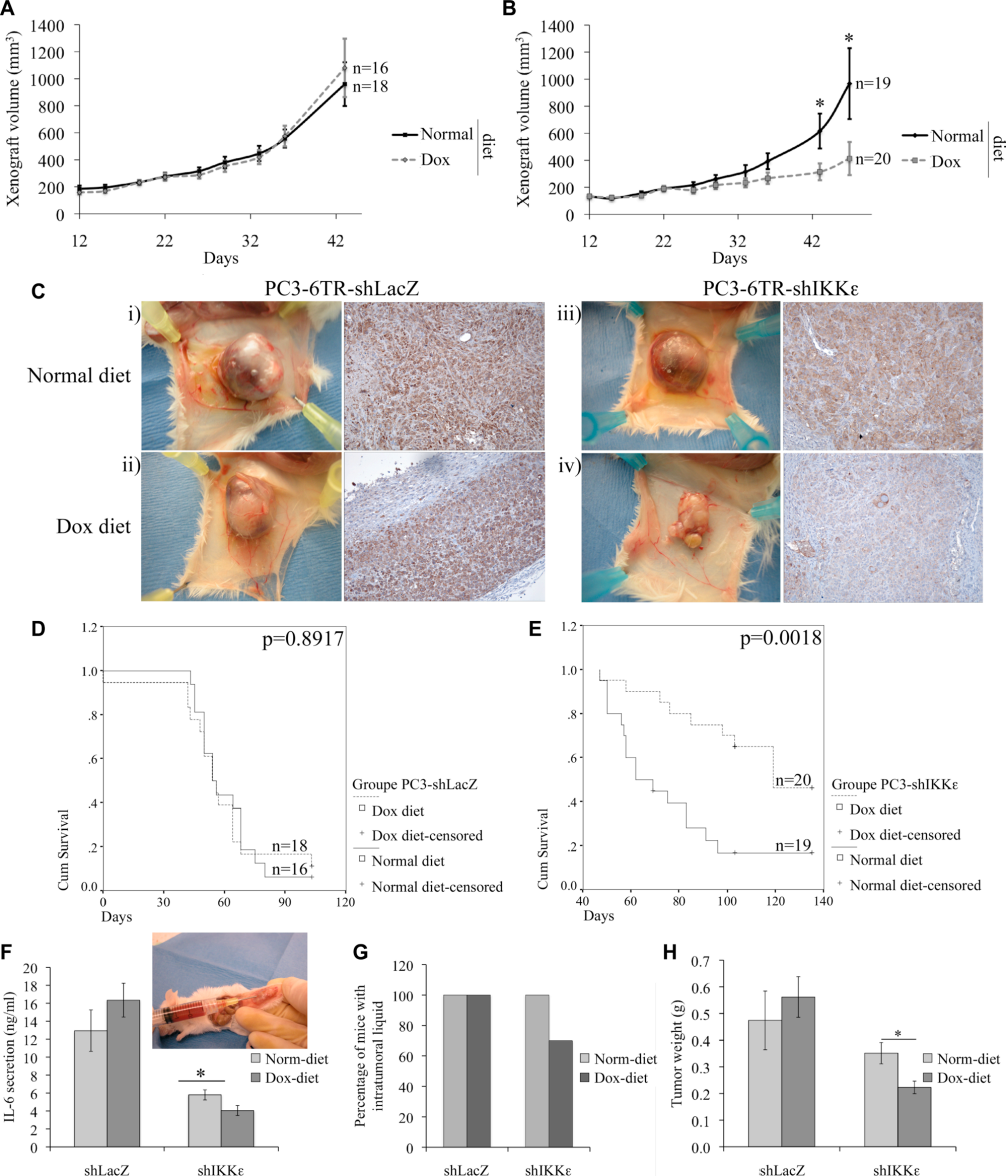
Effect of IKKε depletion on PC-3 tumor growth One million PC3-6TR-shLacZ (**A**) or PC3-6TR-shIKKε (**B**) cells were injected into the left flank of six-week-old male SCID mice fed a normal diet (Normal) or 625 mg/kg doxycycline-supplemented diet (Dox). Tumor size was monitored twice a week. Mice were sacrificed and tumors were collected at 135 days after injection or when the tumor volume reached 2,500 mm^3^. Anti-IKKε immunohistochemical staining (**C**) was performed on paraffin sections prepared from PC3-6TR-shLacZ (i-ii) and PC3-6TR-shIKKε (iii–iv) resected xenografts taken from mice fed normal (i and iii) or doxycycline-supplemented diet (ii and iv). The Kaplan-Meyer curve represents the overall survival of mice injected with PC3-6TR-shLacZ (**D**) and PC3-6TR-shIKKε (**E**) cells. At the last time-point, intratumoral liquids were collected to measure: IL-6 secretion by ELISA (**F**), the percentage of mice with intratumoral liquids (**G**), and tumor weight (**H**). Error bars represent the standard error of the mean. “n” indicates number of mice in each group. *P < 0.05 (paired *t-test*).

Recently, it has been shown that chemical inhibition of TBK1/IKKε dimer activity significantly impairs tumor development in xenograft models [27]. Since we observed an effect of IKKε knock-down on PC-3 cell proliferation (Figure 1F), we tested the effect of IKKε depletion on the growth of PC3 xenografts *in vivo* (Figure 2).

We initiated a large-scale study using 73 mice divided into two cohorts and subcutaneously injected with 1 × 10^6^ PC3-6TR-shLacZ or PC3-6TR-shIKKε cells. Each cohort was randomly subdivided into two groups one week prior to injection and fed either a normal diet or a doxycycline-supplemented diet (dox-diet). We observed a significant delay in the growth of the PC3-6TR-shIKKε tumors depleted for IKKε expression by the dox-diet (Figure 2B-Dox) as compared to the PC3-6TR-shIKKε tumors in mice on a normal diet (Figure 2B-Normal) or to the control PC3-6TR-shLacZ tumors (Figure 2A; Normal and Dox-diet). Immunohistochemistry staining of xenografts using anti-IKKε antibody confirmed the efficiency of the diet-dependent IKKε knock-down (Figure 2C). The constitutive expression of IKKε in LacZ cells was not affected by doxycycline stimulation (Figure 2C-ii). We also noted a small decrease in IKKε staining intensity in the PC3-6TR-shIKKε tumors of mice that were fed a normal diet (Figure 2C-iii). This decrease confirmed the leakiness of our Tet repression system. Since we compared each clone with or without induction rather than comparing the shLacZ and shIKKε clones to each other, the impact of the observed Tet repression leakage should not impact the conclusion of the study.

Kaplan-Meier curves were constructed to illustrate the survival difference (endpoint reached when tumor volume was 2,500 mm^3^) between mice injected with PC3-6TR-shLacZ (Figure 2D) or PC3-6TR-shIKKε (Figure 2E) cells and fed a normal or dox-diet. As expected, no difference between normal and dox-diet was observed in the survival of mice injected with PC3-6TR-shLacZ cells (*P* = 0.8917, Figure 2D). The survival of mice injected with PC3-6TR-shIKKε cells and fed a dox-diet was significantly increased compared to the same mice fed a normal diet (*P* = 0.0018, Figure 2E). Notably, 55% (11/20) of the PC3-6TR-shIKKε injected mice fed the dox-diet were sacrificed at the end of the study (day 135) before the tumor reached the endpoint volume, compared to only 21% (4/19) of mice fed a normal diet. In five out of twenty (25%) PC3-6TR-shIKKε injected mice fed the dox-diet, tumor growth stopped completely after 50 to 60 days (tumor volume between 400 and 500 mm^3^), and did not increase during the rest of the study (final sacrifice at day 135, data not shown). Moreover, in two of these mice, PC3-6TR-shIKKε tumors reached 1,300 and 1,700 mm^3^ at day 98 before regressing to 800 and 1,200 mm^3^ respectively at day 135 (data not shown). Altogether, this shows a significant decrease in the growth of PC3 tumors lacking IKKε.

As most of the PC3-6TR-shLacZ and PC3-6TR-shIKKε tumors formed a fluid pocket, we collected the intratumoral liquid during the necropsy and measured their IL-6 levels by ELISA (Figure 2F). IL-6 secretion levels were much lower in xenografts of PC3-6TR-shIKKε than of PC3-6TR-shLacZ and correlated with IKKε expression. Moreover, for six of the twenty PC3-6TR-shIKKε injected mice fed a dox-diet, no intratumoral liquid was observed (Figure 2C-iv, 2G). To confirm that the differences in tumor volumes (Figure 2A, 2B) were not only due to the quantity of intratumoral fluid, we weighed the tumors after their excision and fluid removal (Figure 2H). We observed no significant effect of dox-diet on the weight of PC3-6TR-shLacZ tumors but a significant decrease in the weight of PC3-6TR-shIKKε tumors from mice fed with the dox-diet.

### Identification of the IL-6 promoter sequences involved in IKKε-dependent activation

Since our previous study demonstrated that IKKε over-expression resulted in an increase of IL-6 secretion in PC cell lines, we examined whether IKKε could modulate cytokine gene transcription in both HS and CR PC cell lines.

**Figure 3:**
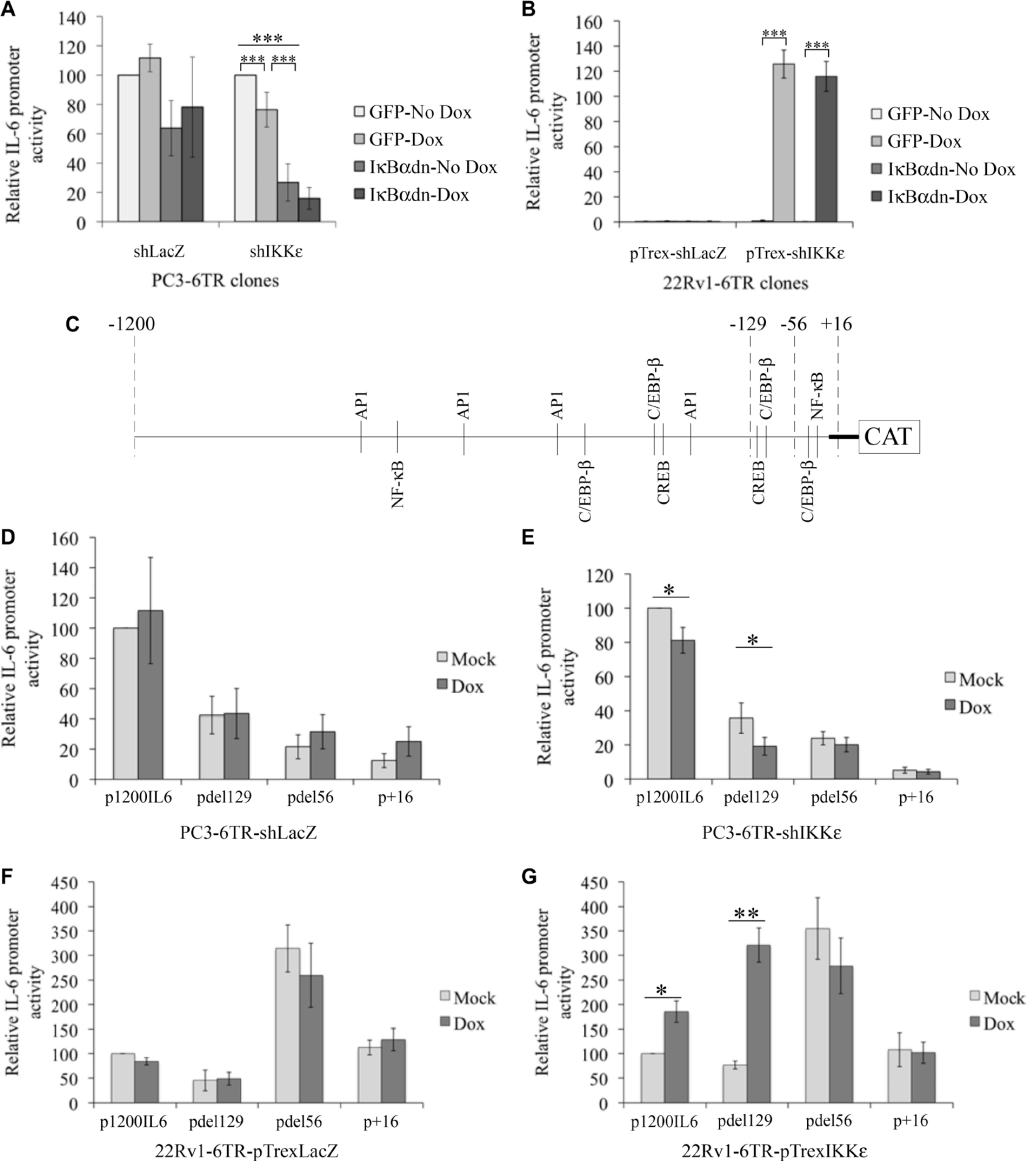
Characterization of IKKε-dependent regulation of IL-6 promoter activity in prostate cancer cell lines (**A**–**B**) Effect of NF-κB inhibition on IKKε-dependent regulation of IL-6 promoter activity. Stimulation of IL-6 promoter was measured by CAT ELISA in PC3-6TR-shLacZ/shIKKε (A) and 22Rv1-6TR-pTrexLacZ/IKKε (b) clones. Cells were co-transfected with p1200IL6-CAT and pCMV-IκBαdn or pCMV-GFP (as a control) constructs. Forty-eight hours later, cells were lysed and assayed for CAT expression by ELISA. CAT protein quantification was adjusted for 1 mg total cell proteins. Each measurement was done in triplicate and each experiment was repeated three times. (**C**) Schematic representation of IL-6 promoter. Each potential TF binding site of the four major TFs involved in IL-6 gene regulation are indicated. (**D**–**G**) Identification of the IL-6 promoter region activated in response to IKKε over-expression in PC3-6TR-shLacZ/shIKKε (D–E) and 22Rv1-6TR-pTrexLacZ/IKKε (F–G) cell lines. Cells were transfected with the p1200IL6-CAT and three truncated IL-6 promoter vectors pdel129-CAT, pdel56-CAT and p+16-CAT. Forty-eight hours later, cells were lysed and assayed for CAT expression by ELISA as described above. Each measurement was done in duplicate on two (22Rv1-6TR-pTrexLacZ/IKKε) or three (PC3-6TR-shLacZ/shIKKε) clones and each experiment was repeated three times. Error bars represent the standard error of the mean. *P < 0.05; **P < 0.001 (paired *t-test*).

To study the effect of IKKε over-expression on IL-6 promoter activity, we generated several doxycycline-inducible 22Rv1-6TR-pTrexIKKε and 22Rv1-6TR-pTrexLacZ clones. Notably, we observed a strong correlation between the levels of IKKε expression and the amount of IL-6 secretion in 22Rv1-6TR-pTrexIKKε clones (Supplementary Figure S2). We selected the 22Rv1-6TR-pTrexIKKε clones 1 and 6 for further studies. Using the p1200IL6-CAT vector (1200 bp upstream of the transcription initiation site cloned in front of the CAT gene [28]), we followed IL-6 promoter activity in our CR and HS PC cell models by CAT ELISA (Figure 3). We found that depletion of IKKε in CR PC-3 cells induced a significant decrease in IL-6 promoter stimulation (Figure 3A, *P* < 0.001, *t-test*). We also observed that IKKε over-expression in 22Rv1 cells led to strong IL-6 promoter activity (Figure 3B). As NF-κB is the major regulator of IL-6 gene expression [28, 29], we studied the effect of NF-κB inhibition on the IL-6 promoter by the transient transfection of the pCMV- IκBαdn construct in addition to IKKε knock-down/over-expression. The IκBαdn is a dominant negative form of the NF-κB inhibitor, IκBα, which cannot be phosphorylated and thereby inhibits the activation of NF-κB. As a control for this experiment, we used the pCMV-GFP plasmid. We found that IKKε-dependent regulation of the IL-6 promoter did not involve the activation of NF-κB (Figure 3A and 3B). In particular, the effect of IKKε over-expression on IL-6 promoter stimulation was unaffected by the inhibition of NF-κB activity by IκBαdn in 22Rv1-6TR-pTrexIKKε cells (Figure 3B). We also observed that inhibition of NF-κB using IκBαdn resulted in a greater decrease in IL-6 promoter activity than IKKε depletion alone in PC-3 cells (Figure 3A).

Therefore, IL-6 promoter activation via IKKε requires other transcription factors (TFs) than NF-κB in PC. Presently, only 4 TFs have been reported to control the IL-6 promoter activity: NF-κB, C/EBP-β, CREB and AP1. The consensus binding sites for these TFs are well characterized [30, 31]. Using the bioinformatics software TESS (Transcription Element Search System from the Computational Biology and Informatics Laboratory, University of Pennsylvania), we localized the principal consensus binding sites for these four TFs in the IL-6 promoter region (Genebank NM_000600.3) (Figure 3C). Three truncated IL-6 promoter constructs (pdel129-CAT, pdel56-CAT, p+16-CAT, Figure 3C) [28] were used in transient transfection assays to identify promoter regions that contain regulatory sites associated with IKKε-dependent IL-6 gene expression. Even if the loss of the -1200 to -129 region dramatically affected the IL-6 promoter activity in PC3-6TR-shIKKε (and in its shLacZ control, Figure 3D and 3E), the effect of IKKε depletion on the constitutive activation of IL-6 promoter was only observed using the p1200IL6-CAT (*P* = 0.031, paired *t-test*) and pdel129-CAT constructs (*P* = 0.01, paired *t-test*). In 22Rv1-6TR-pTrexIKKε cells, we noted a significant increase of IL-6 promoter activity in response to IKKε expression using the p1200IL6-CAT and pdel129-CAT constructs (*P* = 0.031 and *P* = 0.003 respectively, paired *t-test*). We also observed a strong activation of the IL-6 promoter using the pdel56-CAT plasmid, but this activation seemed IKKε-independent. The promoter region between -129 and -56 contains two major consensus TF binding sites, one for C/EBP-β and one for CREB, but no NF-κB binding site (Figure 3C). In the 22Rv1-6TR-pTrexLacZ cells, doxycycline stimulation did not induce any activity in all promoter regions studied (Figure 3F). We also noted that the construct with the very proximal region of the promoter (–56 to +1) containing C/EBP-β and NF-κB binding sites appeared to be constitutively active in 22Rv1-6TR-pTrexIKKε cells and their 22Rv1-6TR-pTrexLacZ controls.

### Identification of the transcription factor activated in response to IKKε over-expression

**Figure 4:**
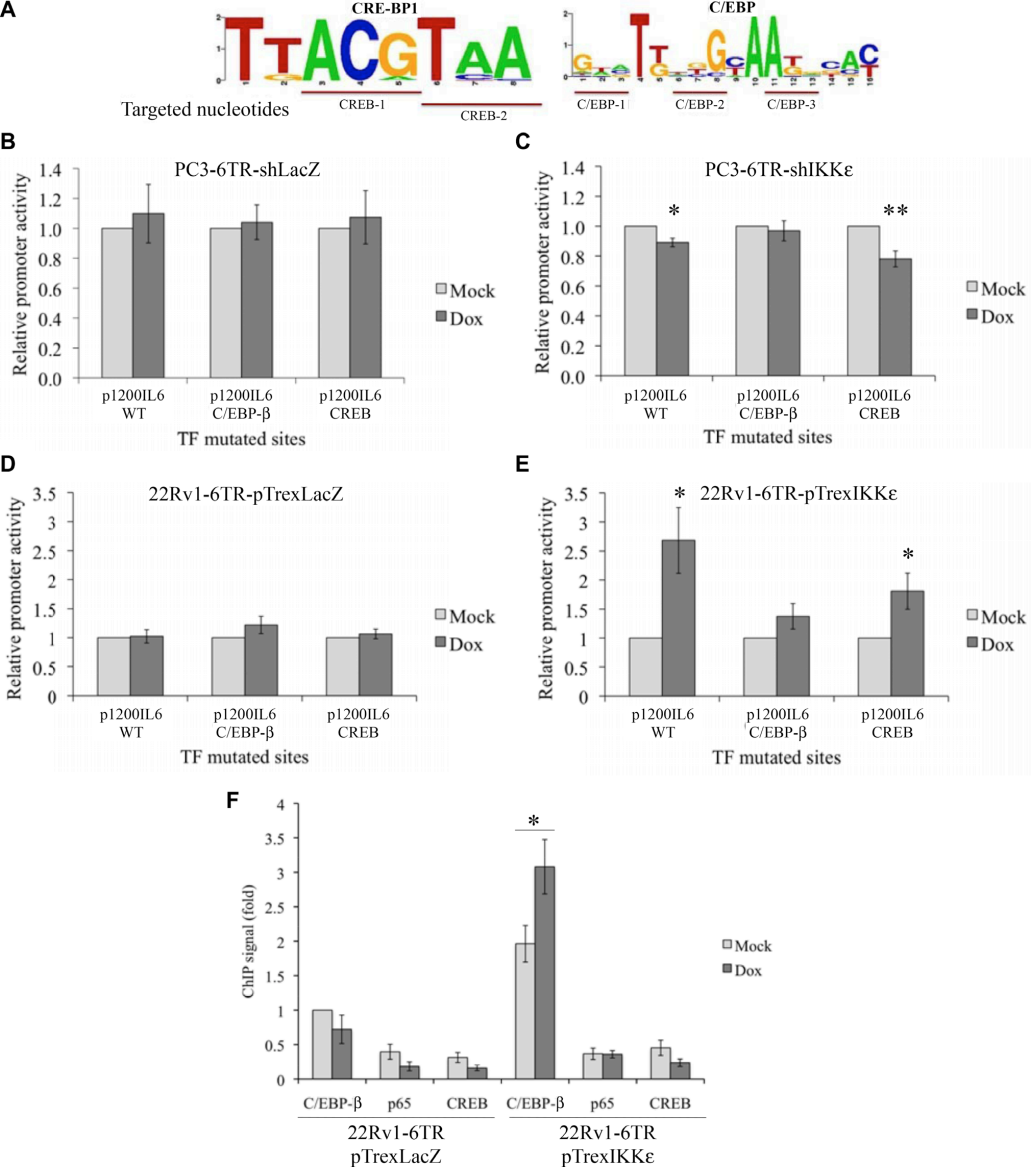
Identification of the IL-6 promoter transcription factor activated in response to IKKε over-expression (**A**) Schematic representation of the most conserved nucleotides in the CREB and C/EBP-β binding sites. (**B**–**E**) Effect of C/EBP-β and CREB binding site mutations on IL-6 promoter activity in PC3-6TR-shLacZ/shIKKε (B, C) and 22Rv1-6TR-pTrexLacZ/pTrexIKKε (D, E) cell lines. Cells were transfected with p1200IL6-CAT or C/EBP-β and CREB constructs with mutated TF binding site. Forty-eight hours later, cells were lysed and assayed for CAT expression by ELISA. CAT protein quantification was adjusted for 1 mg total cell proteins. Each measurement was done in triplicate and each experiment was repeated at least three times. (**F**) TF binding analysis at the IL-6 promoter region. ChIP was performed using anti-p65, anti-CREB and anti-Flag antibodies on cellular extracts from 22Rv1-6TR-pTrexLacZ and 22Rv1-6TR-pTrexIKKε cells that were transiently transfected with pORF9-C/EBP-β-Flag^2^ vector and stimulated with or without doxycycline. Each ChIP signal was calculated relative to the Flag IP signal of non-induced 22Rv1-6TR-pTrexLacZ chromatin preparation. Experiments were done three times on two independent chromatin preparations. *P < 0.05 (paired *t-test*).

To identify the TF activated by IKKε activity, we made several site-directed mutations within the two binding sites located between –129 and –56, CREB (–112 to –105) and C/EBP-β (–105 to –92). Based on the literature [32–34], we identified the most conserved nucleotides in the CREB and C/EBP-β binding sites and performed five different mutagenic variations on the p1200IL6-CAT vector (two for the CREB and three for the C/EBP-β binding sites, Figure 4A).

Using these mutated-p1200IL6-CAT constructs, we followed the effect of IKKε expression on site-mutated IL-6 promoters (Figure 4B–4E) by CAT ELISAs. Mutations in the CREB binding site (–112 to –105) led to a decrease in the IL-6 promoter activity in PC3-6TR-shIKKε (Figure 4C; *P* = 0.003, paired *t-test*) and increase in 22Rv1-6TR-pTrexIKKε (Figure 4E; *P* = 0.025, paired *t-test*) clones. On the other hand, CREB mutations did not abolish the effect of the IKKε knock-down in PC3-6TR-shIKKε, or the impact of IKKε over-expression in 22Rv1-6TR-pTrexIKKε, on IL-6 promoter activity (Figure 4C and 4E). In contrast, the impact of IKKε expression (knockdown or over-expression) on IL-6 promoter activity was lost when the C/EBP-β binding site (–105 to –92) was mutated either in PC3-6TR-shIKKε (Figure 4C; *P* = 0.339, paired *t-test*) or 22Rv1-6TR-pTrexIKKε (Figure 4E; *P* = 0.124, paired *t-test*). As expected, mutations in these CREB and C/EBP-β binding sites did not induce any variation in the IL-6 promoter activity in PC3-6TR-shLacZ (Figure 4B) or 22Rv1-6TR-shLacZ (Figure 4D) clones.

Chromatin immunoprecipitation (ChIP) assays were conducted to assess the interactions between the IL-6 promoter region and the three major IL-6 TFs, NF-κB-p65, CREB and C/EBP-β (Figure 4F), during IKKε expression. To perform this experiment, we transiently transfected 22Rv1-6TR-pTrexIKKε clone 6 and 22Rv1-6TR-pTrexLacZ clone A with a flag-tagged C/EBP-β (Supplementary Figure S3). Dox-induced IKKε showed an increase of IL-6 endogenous promoter region occupancy by C/EBP-β in 22Rv1-6TR-pTrexIKKε cells compared to the control (3.3 times), whereas no difference was found between controls and IKKε over-expressing cells for NF-κB/p65 and CREB (Figure 4F). These results suggest that IKKε regulates IL-6 promoter activity via activation of the TF C/EBP-β.

### IKKε binds to C/EBP-β and induces its phosphorylation and nuclear translocation

**Figure 5:**
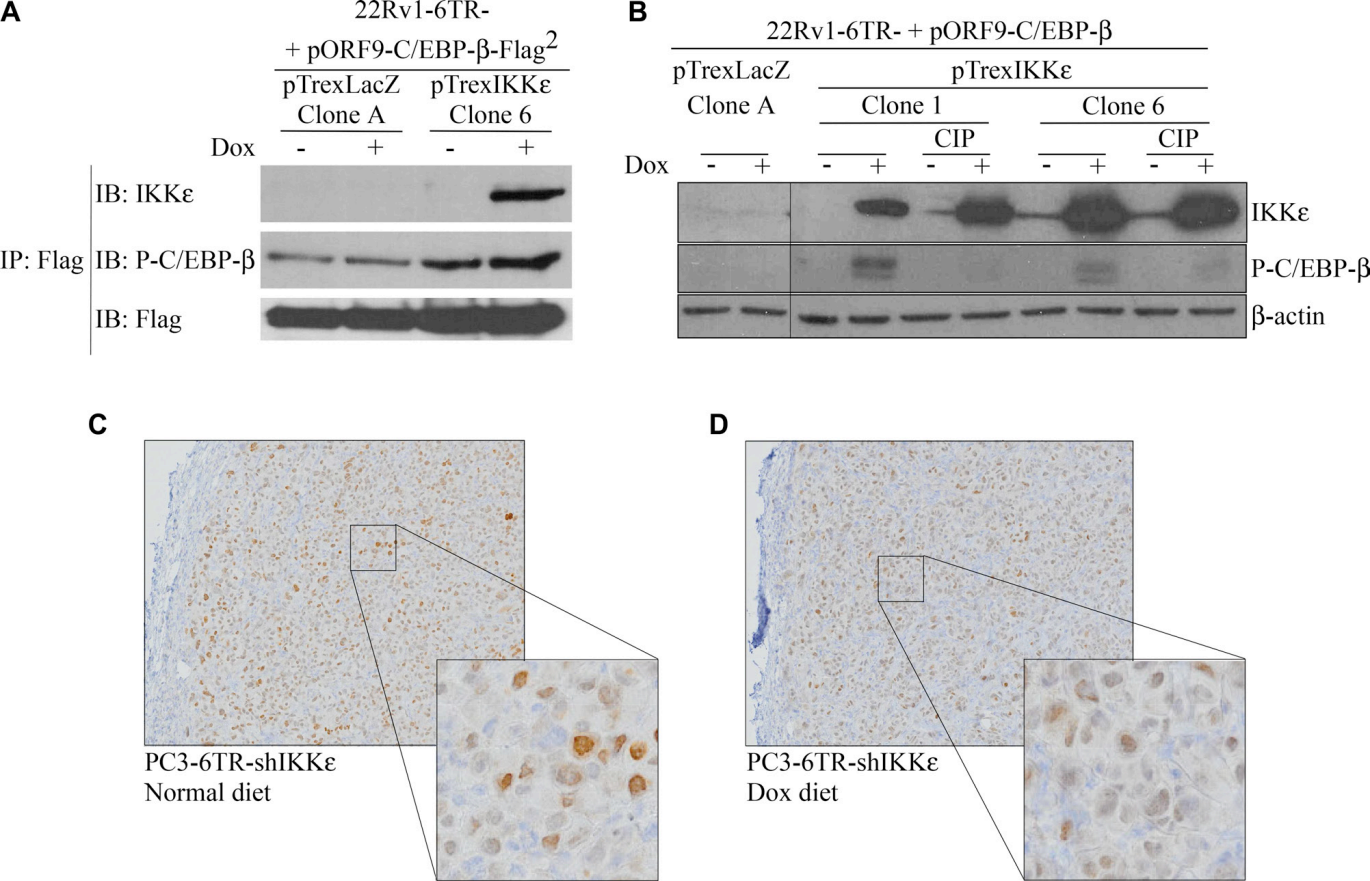
Activation and nuclear translocation of C/EBP-β is induced by IKKε binding (**A**) Co-immunoprecipitation of IKKε and C/EBP-β. Cells of 22Rv1-6TR-pTrexLacZ and 22Rv1-6TR-pTrexIKKε were transfected with pORF9-C/EBP-β-Flag^2^ vector and stimulated with doxycycline. Immunoprecipitation using an anti-Flag antibody was performed and proteins were resolved on SDS-PAGE, transferred onto PVDF, and probed with anti-IKKε and anti-phospho-C/EBP-β antibodies. (**B**) Correlation between IKKε over-expression and C/EBP-β phosphorylation in 22Rv1-6TR-pTrexLacZ (clone A) and 22Rv1-6TR-pTrexIKKε (clones 1 and 6) cells. Cells were transfected with a pORF9-C/EBP-β vector to increase the amount of detec/EBP-β. Whole cell extract proteins were treated with or without a calf intestinal alkaline phosphatase (CIP), resolved by SDS-PAGE, transferred onto PVDF and probed with anti-IKKε and anti-phospho-C/EBP-β antibodies. Equal loading was verified with an anti-β-actin antibody. (**C**–**D**) Immunohistochemical staining with anti-C/EBP-β antibody of paraffin sections prepared from PC3-6TR-shIKKε resected xenografts from mice fed normal (C) or doxycycline-supplemented (D) diet. (*P < 0.05; **P < 0.001 (paired *t-test*).

To determine if IKKε directly binds to the TF C/EBP-β in 22Rv1-6TR-pTrex IKKε cells, we immunoprecipitated C/EBP-β-associated molecules using an anti-Flag antibody. We observed that IKKε co-immunoprecipitated with C/EBP-β and its phosphorylated form (Figure 5A). No specific bands were obtained using control beads without antibody (data not shown).

Since TF activation is generally achieved by phosphorylation, we followed the effect of IKKε over-expression on C/EBP-β phosphorylation in 22Rv1-6TR-pTrexIKKε clones (Figure 5B). Doxycycline-dependent induction of IKKε expression was correlated with the phosphorylation of C/EBP-β, and this phosphorylation was not detected after sample treatment with calf intestinal alkaline phosphatase (CIP), a phosphoserine and phosphothreonine phosphatase [35].

Finally, previous studies have shown that the phosphorylation of C/EBP-β at Ser^299^ is critical for its nuclear translocation and its subsequent gene transactivation [36–38]. When we analyzed the nuclear accumulation of C/EBP-β by immunohistochemistry in PC3-6TR-shIKKε xenografts from mice fed normal diet (Figure 5C) or dox-diet (Figure 5D), we observed a significant decrease of C/EBP-β nuclear staining when IKKε was depleted from the tumor. Inhibition of IL-6 expression using siRNA did not affect the IKKε-dependent phosphorylation and nuclear translocation of C/EBP-β (Supplementary Figure S3).

## DISCUSSION

A proposed target for the treatment of CR PC patients is IL-6 [39, 40], an inflammatory cytokine that has been associated with CR progression [41], development of bone metastasis [42] and metastasis-related morbidity [5, 6]. We have previously demonstrated a strong correlation between the over-expression of IKKε and IL-6 secretion in PC cell lines [18]. We have also shown the biological link between IKKε and PC progression *in vivo* by analyzing IKKε expression in a cohort of 141 PC patients [22]. In this previous study, we found an increase in cytoplasmic IKKε expression during PC progression, increasing from non-malignant tissues to CR PC. In the present study, we demonstrate the role of IKKε as an oncogene in PC and detail the molecular mechanism involved in the regulation of IL-6 expression, activated in response to IKKε over-expression.

Considering the inhibitory effect of IL-6 knock-down on PC-3 cell growth *in vitro* (Figure 1A and 1B; [43, 44]) and *in vivo* [44, 45], and the importance of IKKε in the regulation of IL-6 secretion in PC, we studied the role of IKKε on PC-3 cell proliferation and tumor formation. In line with our previous results [18], depletion of IKKε expression resulted in a substantial decrease (> 50%) in IL-6 secretion by PC-3 cells (Figure 1D) and a decrease in cell proliferation *in vitro* (Figure 1F). This inhibitory effect of IKKε knock-down on cell proliferation has previously been observed *in vitro* in HeLa, ovarian and breast cancer cells [19, 21, 46]. We also showed the impact of IKKε depletion *in vivo* via PC-3 tumor formation and growth in SCID mice (Figure 2). In particular, 135 days after PC-3 cell injection, we observed that 35% of mice injected with IKKε depleted cells displayed a complete arrest of tumor growth and even a reduction in tumor mass (Figure 2E, 2H). These observations were in line with recent studies in which the authors showed that intratumoral delivery of synthetic IKKε siRNA interfered with subcutaneous glioma growth in Nude mice [47], and that treatment with TBK1/IKKε inhibitors significantly impaired development of human squamous cell carcinoma in these mice [27]. Together, these studies suggest a more general role for IKKε in cancer.

During mouse necropsies, we observed that all PC3-6TR-shLacZ and PC3-6TR-shIKKε tumors in mice fed a normal diet displayed the formation of a fluid pocket around the tumor mass (Figure 2F). We analyzed these intratumoral liquids and recovered a very high concentration of IL-6 (PC3-6TR-shLacZ normal diet: 13 ng/ml; PC3-6TR-shLacZ dox-diet: 16.3 ng/ml; PC3-6TR-shIKKε normal diet: 5.8 ng/ml) and IL-8 (218 ng/ml; 257 ng/ml; 163 ng/ml respectively). To our knowledge, formation of inflammatory fluid pockets around subcutaneous xenografts has never been reported before for wild-type PC-3 xenografts. The appearance of this fluid pocket could be in response to the high level of inflammatory cytokine secretion in PC3-6TR-shLaZ/shIKKε clones as previously described *in vitro* [18]. Seventy percent of PC3-6TR-shIKKε tumors in mice fed a dox-diet presented a similar pocket with an average concentration of IL-6 and IL-8 (4 ng/ml and 118 ng/ml respectively) that was significantly lower. The remaining tumors (30%), which did not form an inflammatory fluid pocket, completely stopped growing. This is in line with the fact that in PC cell lines, IL-6 stimulates cell growth in an autocrine and paracrine manner and is involved in PC development and progression [12, 43, 48, 49]. To confirm the impact of IKKε-depletion, not only on the intratumoral cytokine secretion but also on tumor growth, we weighed each individual xenograft during necropsies and observed a large reduction in tumor mass when IKKε was depleted (Figure 2H). We conclude from these experiments that IKKε has a pro-proliferative role in the CR PC-3 cell line, and this role is in part, due to the activation of IL-6 expression.

To investigate which TF is responsible for IKKε-dependent IL-6 promoter induction, we took advantage of the IL-6 promoter constructs already generated by Sanceau *et al*. [28] to characterize the activity of the proximal IL-6 promoter region. Using these constructs, we confirmed the major role of NF-κB on the constitutive activation of the IL-6 promoter in PC-3 cells (Figure 3A) but we showed that the IKKε-dependent IL-6 promoter activity, particularly in 22Rv1 cells, is NF-κB-independent (Figure 3A and Figure 1B, Figure 4F). As NF-κB alone is unable to fully activate IL-6 gene transcription, and requires additional TFs to induce complete activation [50, 51], we analyzed the 1200 bp promoter region for the presence of TF binding sites using TESS. Notably, this bioinformatics software did not identify a consensus IRF-3/−7 regulatory sequence in this promoter region, confirming our previous results which showed that IKKε-dependent IL-6 secretion does not involve IRF-3 or IRF-7 activation [18]. We found that the deletion of the -1200 to -129 region reduces IL-6 promoter activity but only the deletion of the –129 to –56 region completely abolished IKKε-dependent induction of the IL-6 promoter in both cell lines studied (Figure 3). Subsequently, we concentrated our study on the –129 to –56 region, which contains two consensus TF binding sites, one for CREB and one for C/EBP-β (Figure 3C). Only mutations in the C/EBP-β binding site (–105 to –92 region) completely abolished the IKKε-dependent regulation of the IL-6 promoter activity in 22Rv1-6TR-pTrexIKKε and PC3-6TR-shIKKε clones (Figure 4B–4E) and IKKε over-expression only induced the C/EBP-β binding to the endogenous IL-6 promoter region (Figure 4F). In PC-3 cells, a previous study showed that over expression of C/EBP-β or NF-κB induced IL-6 promoter activity but the strongest induction occurred with the simultaneous over-expression of both TFs and was largely dependent upon an intact C/EBP-β binding site localized between –144 and –132 [52]. This TF binding site is the same as the one we identified and mutated in the –129 to –56 region, the difference in the localization (–144 to –132 compared to –105 to –92) is due to a recent reposition of the IL-6 gene transcription start site (Genebank NM_000600.3).

C/EBP-β is a widely expressed TF involved in proliferation, terminal differentiation and growth arrest in different cell types [53]. Numerous studies have previously shown that C/EBP-β activity affects several aspects of PC disease progression. This TF can act as co-repressor of AR in PC [54, 55], regulates the expression of steroidogenic genes [56, 57], and participates in the regulation of metastatic and PC cell survival genes [58, 59]. In this study, we observed that C/EBP-β expression levels increased in PC3-6TR-shIKKε xenografts expressing IKKε compared to xenografts depleted for IKKε (Figure 5C–5D). It was previously reported that C/EBP-β levels are significantly higher in PC samples from CR patients compared with therapy-naïve patients, and upon androgen deprivation, C/EBP-β mRNA and protein expression are rapidly increased in HS PC cell lines [60]. Moreover, its expression seems directly regulated by AR activity as AR can bind to and suppress the C/EBP-β proximal promoter [60]. These observations suggest that C/EBP-β expression may correlate with the IKKε expression previously described in PC [17, 22]. In these studies, we showed that IKKε is constitutively expressed in CR PC cell lines compared to HS cell lines in which its expression is inducible and AR-dependent [17]. We also observed that CR PC tissues present the highest cytoplasmic IKKε expression levels, and found a strong link between increased IKKε cytoplasmic expression and metastatic progression [22]. Since phosphorylation plays an important role in regulating C/EBP-β nuclear translocation and its subsequent gene transactivation [36, 61], we analyzed the impact of IKKε expression on the phosphorylation of C/EBP-β. We found that IKKε directly interacted with C/EBP-β (Figure 5A) and IKKε over-expression induces not only C/EBP-β phosphorylation (Figure 5B) but also its nuclear accumulation in PC cells (Figure 5C). In contrast, we failed to detect any phosphorylation of CREB when IKKε was over-expressed (data not shown).

In summary, our study demonstrates that IKKε has a pro-proliferative role in CR PC cell lines, achieved through the phosphorylation and nuclear translocation of C/EBP-β, which promotes the initiation of IL-6 gene expression.

## MATERIALS AND METHODS

### Cell lines, slones and cell culture

HS 22Rv1 and CR PC-3 cells were purchased from the American Type Culture Collection (ATCC CRL-2505 and ATCC CRL-1435 respectively; Manassas, VA). Cells were grown in RPMI 1640 (Wisent, Inc. St-Bruno, Quebec, Canada) supplemented with 100 μg/ml gentamicin, 0.25 μg/ml amphotericin B (Invitrogen, Paisley, United Kingdoms) and 10% fetal calf serum (FCS). In order to modulate IKKε expression in these cell lines, we engineered 22Rv1-6TR-pTrexIKKε cells in addition to previously described PC3-6TR-shIKKε cells [18] (and their respective 22Rv1-6TR-pTrexLacZ/PC3-6TR-shLacZ controls). For the construction of the pT-Rex-DEST30-IKKε vector (abbreviated pTrex-IKKε), we amplified the IKKε gene by PCR (primers described in Supplementary Table S1) using the pUNO-hIKKε plasmid (Invitrogen) as template and cloned it under the control of a tetracycline-inducible promoter in the pT-Rex™-DEST30 vector (Invitrogen). We stably transformed 22Rv1 cells with pcDNA6/TR (Invitrogen) using Lipofectamine 2000 reagent (Invitrogen) according to the manufacturer's instructions. A 22Rv1-pcDNA6/TR clone over-expressing TetR (abbreviated 22Rv1-6TR) was selected and used to create stable 22Rv1-6TR-pTrexIKKε cell lines. The 22Rv1-6TR cells were transfected with the pTrex-IKKε construct or with pT-Rex/GW-30/LacZ vector (Invitrogen) and selected in RMPI with 10% FCS supplemented with 1 μg/ml Zeocin (Invitrogen). Six stable 22Rv1-6TR-pTrexIKKε clones that differentially over-expressed IKKε, and two stable 22Rv1-6TR-pTrexLacZ clones that over-expressed LacZ were isolated for this study.

### siRNA transfection

Three independent siRNAs targeting IL-6, and the RISC-free siGLO fluorescent siRNA control were purchased from Dharmacon (Chicago, IL). Transient transfection of PC-3 cells with IL-6-targeting siRNAs or siGLO was performed as recommended by the manufacturer using DharmaFECT 2 transfection reagent (Dharmacon). Briefly, cells were seeded at 60 000 cells per well on 12-well plates and allowed to grow for 24 hours prior to transfection. Cells were then transfected with 25 nM siRNA for 24 hours. The following day, transfection media was replaced by low-serum culture medium (RPMI 1640 supplemented with 1% FCS). Five days post-transfection, supernatants were collected to measure IL-6 secretion by ELISA and cells were counted using a hemocytometer. Experiments were repeated four times in triplicate.

### Cell proliferation

Cells were plated at a density of 50 000 cells per well in six-well plates and grown in 2 ml of low-serum culture medium (FCS 2%). One, three, five and seven days following plating, cells were washed with PBS and incubated in 500 μl trypsin at 37°C for 10 minutes. Cells were then counted with a hemocytometer. Experiments were repeated four times in triplicate.

IKKε was silenced in PC3-6TR-shIKKε cells for 4 days before the beginning of the exogenous IL-6 rescue assay. Cells were then transferred to a 96-well plate (6000 cells/well) and incubated with or without IL-6 (50 ng/mL). Cell confluence was assessed using an IncuCyte™ live cell monitoring system (Essen BioScience, Ann Arbor, MI) for 5 days. Frames were captured at 2-hour intervals using a 10× objective. Data were expressed as the mean fold change relative to cell confluence in the first picture.

### Western blot analysis

For Western blot analysis, 20–50 μg of proteins from whole cell extracts were resolved on 7.5–12.5% SDS bis-polyacrylamide gels and then transferred onto polyvinylidene difluoride (PVDF) membranes. Whole cell extracts were obtained after 30 minutes incubation in lysis buffer [1% Igepal CA630 (Sigma-Aldrich St. Louis, MI), 10% glycerol, 50 mM Tris, 2 mM EDTA, and 150 mM NaCl] supplemented with freshly added protease and phosphatase inhibitors [(protease inhibitor cocktail from Roche Applied Science, Penzberg, Germany), 5 mM NaF, 200 μM Na_3_VO_4_, and 100 μM PMSF). Anti-IKKε antibody (IMG-5571) was purchased from IMGENEX (San Diego, CA) while the anti-phospho-C/EBP-β antibody (#3084) was obtained from Cell Signaling Technology (Danvers, MA). To ensure equal protein loading, PVDF membranes were probed with an anti-β-actin antibody (ab6276, Abcam, Cambridge, UK).

### Prostate cancer xenograft models

Six-week-old male NOD SCID mice were purchased from Charles River Laboratories (Montreal, QC, Canada) and were maintained on site at the CRCHUM animal facilities. All mouse experiments were approved and performed according to the Comité Institutionnel de la Protection des Animaux (CIPA) animal care guidelines. Doxycycline-inducible PC3-6TR-shIKKε clones, as well as PC3-6TR-shLacZ control clones, were injected into the flank of male NOD SCID mice (1 × 10^6^ cells per mouse). Cells were mixed with matrigel (v/v) prior to injection to limit their spread and to allow the formation of denser, better defined tumors. To minimize stress in mice, doxycycline was delivered through their diet with doxycycline-supplemented food (625 mg/kg; Harlan Laboratories, Indianapolis, IN). Doxycycline-supplemented diet (dox-diet) was started one week prior to PC cell injection. During experiments, no dehydration or weight loss was observed in mice fed with the dox-diet. Tumor volumes were calculated using the following equation: V (mm^3^) = a × b × h, where a is the largest diameter, b is the perpendicular diameter and h is the height. Tumor size was monitored twice a week until tumors reach 2500 mm^3^. At this endpoint, intratumoral liquid was collected (if present) for IL-6 quantification by ELISA, and tumors were excised from sacrificed mice. Tumor mass was weighed, sectioned into small pieces, fixed in formalin and mounted in paraffin blocks. Tissue sections were subsequently probed for IKKε with the anti-IKKε (CT) antibody (dilution 1/300) from ProSci Incorporated (Poway, CA) using a biotin-streptavidin peroxidase method as previously described [62]. Kaplan-Meier tests were performed using SPSS software, version 16 (SPSS, Inc., Chicago, IL).

### Measurement of IL-6 secretion and IL-6 promoter stimulation by ELISA

IL-6 promoter stimulation was measured by Chloramphenicol Acetyl Transferase (CAT) ELISAs with whole cell extracts collected 40 hours after transient transfection of 22Rv1-6TR-pTrexIKKε/LacZ and PC3-6TR-shIKKε/shLacZ cells with p1200IL6-CAT and three truncated IL-6 promoter vectors (originally named pdel36-CAT, pdel108-CAT and pdel189-CAT) kindly provided by Dr. Wietzerbin [28]. According to the present IL-6 gene nomenclature from Genebank (NM_000600.3), which positions the transcription start site 52 nucleotides upstream of the putative transcription start site initially described [28], we now refer to these truncated IL-6 promoter constructs as follow: p+16-CAT, pdel56-CAT and pdel129-CAT respectively. For the measurement of IL-6 secretion, cell supernatants were filtered using 0.2 μm syringe filters and stored at –20°C until use. Remaining cells were scraped and proteins were extracted for CAT ELISA assays. IL-6 secretion in culture media or intratumoral liquid, and CAT concentration in whole cell extracts were measured according to the manufacturer's conditions using DuoSet ELISA kits from R&D Systems (Minneapolis, MN) or CAT ELISA kits from Roche Diagnostics (Mannheim, Germany) respectively. The detection limit was 9.38 pg/ml for IL-6 and 50 pg/ml for CAT. IL-6 secretion and CAT expression were standardized by total protein concentration of each sample as measured by Bradford assay. All assays were carried out in triplicate and each experiment was repeated three to six times.

### Site-directed mutagenesis of -129 to -56 within the IL-6 promoter region

*In vitro* site-directed mutagenesis was performed on p1200IL6-CAT vector using the QuikChange XL system from Agilent Technologies (Santa Clara, CA) according to the manufacturer's instructions. Due to its higher-fidelity, Phusion DNA polymerase from Finnzymes (Espoo, Finland) was utilized instead of the polymerase proposed in the QuikChange XL kit. Mutated constructs were verified by sequencing. Primers used to create point mutations in CREB and C/EBP-β binding sites (Figure 4A) are described in Supplementary Table S1.

### Construction of the pORF9-C/EBP-β-Flag^2^ vector

pORF9-C/EBP-β was obtained from InvivoGen (San Diego, CA). The pORF9-C/EBP-β-Flag^2^ vector was derived from pORF9-C/EBP-β by insertion of a lab-designed double-stranded oligonucleotide containing two Flag sequences (Flag^2^, Supplementary Table S1) and flanked by Sph I restriction sites for cloning. This double-stranded oligonucleotide was then cloned into the 3′ region of the C/EBP-β gene of the pORF9-C/EBP-β plasmid. Plasmids were verified by sequencing.

### Binding of transcription factors to the IL-6 promoter

Chromatin ImmunoPrecipitation (ChIP) assay was carried out with the Upstate Cell Signaling kit (#9003, Cell Signaling Technology). Cells were plated in four 150 mm dishes and transfected with the pORF9-C/EBP-β-Flag^2^ vector using Lipofectamine 2000 reagent (Invitrogen) according to the manufacturer's instructions. After 48 hour of doxycycline or mock treatment, chromatin was prepared accordingly to the manufacturer protocol. Cells were cross-linked with 1% formaldehyde for 10 minutes at 37°C. Cross-linking was terminated with 1× Glycine for 5 minutes at room temperature. Cells were then washed twice in PBS and lysed with 10 ml 1× Buffer A. Nuclei were lysed in 1 ml 1× buffer B and DNA was incubated with 5 μl of Micrococcal nuclease for 20 minutes at 37°C. The reaction was stopped with 0.05 M EDTA. Nuclei pellet were suspended in 1 ml 1× CHIP buffer and sonicated 4 pulses (10–15 sec) to generate DNA fragments that were 200–1,000 base pairs in length. Cross-linked chromatin was precipitated by centrifugation and used for further immunoprecipitation. For each immunoprecipitation, 10–15 μg of chromatin was incubated O/N at 4°C with 2 μg of the following antibodies: anti-Flag (F1804, Sigma), anti-p65 (sc-8008, Santa Cruz Biotechnology Inc., Santa Cruz, CA), anti-CREB (#9197, Cell Signaling Technology), and anti-histone H3 as positive control or normal rabbit IgG to account for non specific binding to the beads. The next day, precipitation was carried out with 30 μl magnetic beads for 2 hours at 4°C. After 3 low salt washes and 1 high salt wash, chromatin was eluted at 65°C for 30 minutes in 150 μl of CHIP elution buffer. Cross-linking was reversed at 65°C for 2 hours by addition of 6 μl of 5 M NaCl and 2 μl proteinase K solution. Precipitated DNA was purified using DNA purification columns provided in the kit. Quantitative PCR using KAPA SYBR fast qPCR kit (Kapa Biosystems, Wilmington, MA) was performed on 2 μl of immunoprecipitated DNA using the IL-6 ChIP primers (Supplementary Table S1). Specific C/EBP-β, CREB and κB sites were amplified in the IL-6 gene promoter, and normalized to input cross-linked DNA for each sample.

### IKKε-C/EBP-β-Flag^2^ co-immunoprecipitation assay

Briefly, 2 μg of anti-Flag M2 (F1804, Sigma-Aldrich) or anti-IKKε (IMG-5571, Imgenex) antibodies were diluted in lysis buffer and incubated with Dynabeads^®^ Protein G (Life Technologies) for 10 minutes at room temperature. After PBS washing, 350 μg of whole cell extract proteins in 200 μl lysis buffer were immunoprecipitated using the Dynabeads-antibody complexes overnight at 4°C under gentle agitation. After PBS washing, protein complexes were eluted using loading buffer (50 mM Tris pH6.8, 10% SDS, 10% glycerol, β-mercapto-ethanol and bromophenol blue) 5–10 minutes at 95°C. Protein complexes were loaded onto 10% polyacrylamide gels and transferred into PVDF membrane.

## SUPPLEMENTARY MATERIALS FIGURES AND TABLES


