# Supplementary material for: IκB-Kinase-epsilon (IKKε) over-expression promotes the growth of prostate cancer through the C/EBP-β dependent activation of IL-6 gene expression
Source: Oncotarget. 2016 Aug 26;8(9):14487–501. doi: 10.18632/oncotarget.11629 (PMC5362420; doi:10.18632/oncotarget.11629)
Supplement: Supplementary file 1 [file oncotarget-08-14487-s001.pdf]

IκB-Kinase-epsilon (IKKε) over-expression promotes the growth of prostate cancer through the C/EBP-β dependent activation of IL-6 gene expression

Supplementary Materials

Supplementary Table S1: Primers used for IKKε gene cloning, mutation of IL-6 promoter transcription binding sites, generation of the pORF9-C/EBP-β-Flag² plasmid and ChIP assays

		Primer sequences
IKKε	Forward	CACCGGTCAGATGCAGAGCACA
	Reverse	GCTCAGACATCAGGAGGT
CREB-1	Forward	CTTGCCATGCTAAAGtgtGTCACATTGCACAATCTTAATAAGG
	Reverse	CCTTATTAAGATTGTGCAATGTGACacaCTTTAGCATGGCAAG
CREB-2	Forward	CTTGCCATGCTAAAGGACGTtggATTGCACAATCTTAATAAGG
	Reverse	CCTTATTAAGATTGTGCAATccaACGTCCTTTAGCATGGCAAG
CEBP-1	Forward	GCTAAAGGACGTCgagTTGCACAATCTTAATAAGGTTTCC
	Reverse	GGAAACCTTATTAAGATTGTGCAActcGACGTCCTTTAGC
CEBP-2	Forward	GCTAAAGGACGTCACATTttcCAATCTTAATAAGGTTTCC
	Reverse	GGAAACCTTATTAAGATTGgaaAATGTGACGTCCTTTAGC
CEBP-3	Forward	GCTAAAGGACGTCACATTGCACAggaTAATAAGGTTTCC
	Reverse	GGAAACCTTATTAAtccTGTGCAATGTGACGTCCTTTAGC
Flag²-SphI	Forward	GATTACAAGGATGACGACGATAAGCACATG
	Reverse	TGCTTATCGTCGTCATCCTTGTAATCCATG
IL-6 ChIP	Forward	GTGTCTTGCCATGCTAAAG
	Reverse	AGACATCTCCAGTCCTATATT

Primers were described as follows: 5′–3′, mutations were written in lower case and underlined.

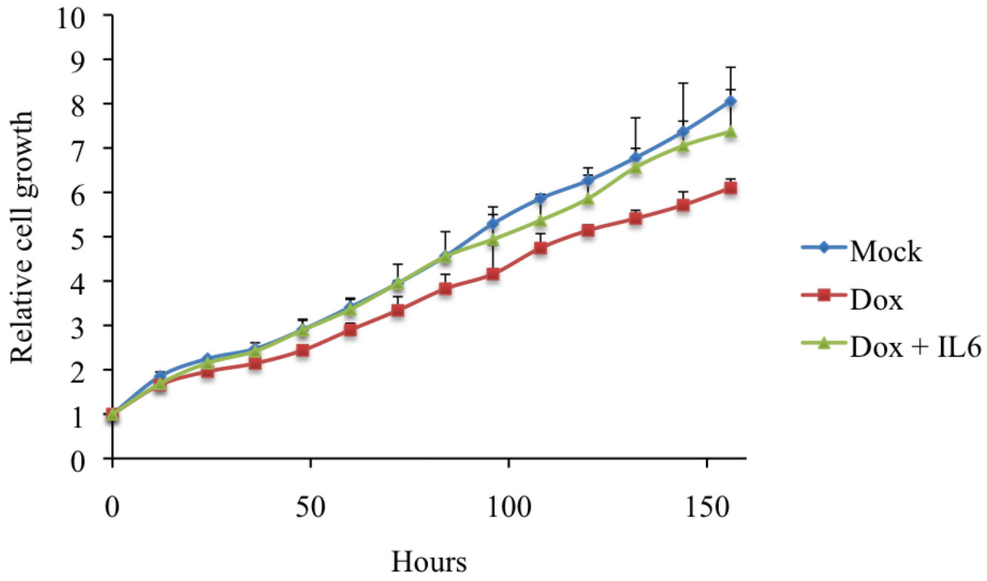

Supplementary Figure S1: The IKKε effect on PC3 cell proliferation after addition of exogenous IL-6. Exogenous IL-6 (50 ng/mL) was added to the low-serum culture medium of PC3-6TR-shIKKε cells. Cell growth was measured using the real-time IncuCyte™ cell proliferation assay (Essen BioScience Inc.). Each measurement was performed in triplicate and each experiment was repeated twice. Error bars represent the standard error of the mean.

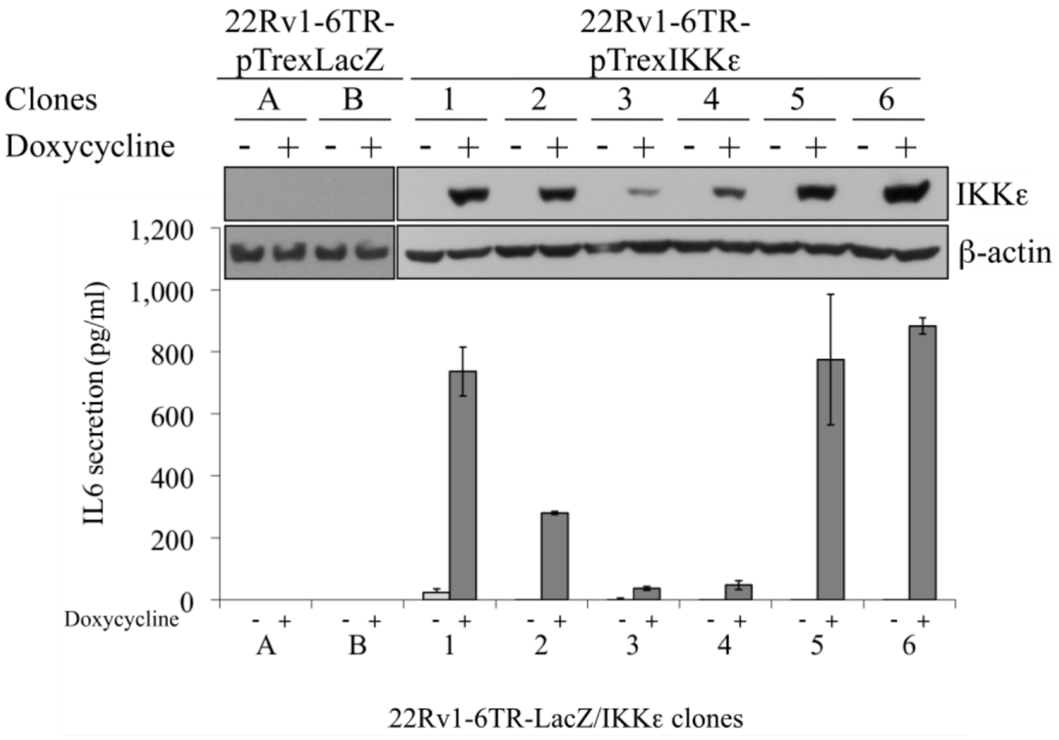

Supplementary Figure S2: Correlation between IKKε expression and IL-6 secretion in 22Rv1-6TR-pTrexLacZ/IKKε clones. Immunoblots with anti-IKKε antibody of whole cell extracts from 22Rv1-6TR-pTrexIKKε and 22Rv1-6TR-pTrexLacZ clones stimulated with doxycycline were compared with IL-6 secretion from each individual clone. For ELISAs, sample concentration was calculated using standard curves. Cytokine secretion was adjusted for 1 ml of cell culture supernatant and for 1 mg total cell proteins. Each measurement was done in triplicate and each experiment was repeated three times. Error bars represent the standard error of the mean.

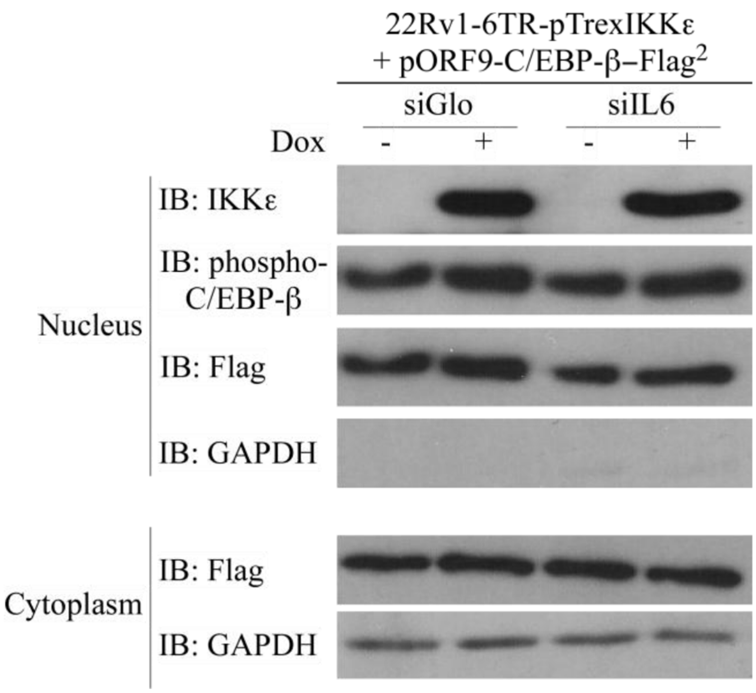

Supplementary Figure S3: Effect of IL-6 silencing on the IKKε-dependent activation of C/EBP-β in 22Rv1-6TR-pTrexIKKε cells. Cells were co-transfected with a pORF9-C/EBP-β-Flag^2 vector and a control siRNA (si-Glo) or a cocktail of three different IL-6 siRNAs (5 μM) prior to doxycycline induction. Two days later, cells were collected and fractionated. Nuclear and cytosolic fractions were resolved by SDS-PAGE, transferred onto PVDF and probed with anti-IKKε, anti-phospho-C/EBP-β and anti-Flag antibodies. Equal loading and cytoplasmic contamination of nucleus extracts were verified with an anti-GAPDH antibody.
